# Bacterial Cell Wall Precursor Phosphatase Assays Using Thin-layer Chromatography (TLC) and High Pressure Liquid Chromatography (HPLC)

**DOI:** 10.21769/BioProtoc.2761

**Published:** 2018-03-20

**Authors:** Manuel Pazos, Christian Otten, Waldemar Vollmer

**Affiliations:** Centre for Bacterial Cell Biology, Institute for Cell and Molecular Biosciences, Newcastle University, Newcastle upon Tyne, UK

**Keywords:** Lipid II, Undecaprenyl pyrophosphate, Phosphatase activity, Peptidoglycan, HPLC, TLC

## Abstract

Peptidoglycan encases the bacterial cytoplasmic membrane to protect the cell from lysis due to the turgor. The final steps of peptidoglycan synthesis require a membrane-anchored substrate called lipid II, in which the peptidoglycan subunit is linked to the carrier lipid undecaprenol via a pyrophosphate moiety. Lipid II is the target of glycopeptide antibiotics and several antimicrobial peptides, and is degraded by ‘attacking’ enzymes involved in bacterial competition to induce lysis. Here we describe two protocols using thin-layer chromatography (TLC) and high pressure liquid chromatography (HPLC), respectively, to assay the digestion of lipid II by phosphatases such as Colicin M or the LXG toxin protein TelC from *Streptococcus intermedius*. The TLC method can also monitor the digestion of undecaprenyl (pyro)phosphate, whereas the HPLC method allows to separate the di-, mono- or unphosphorylated disaccharide pentapeptide products of lipid II.

## Background

The peptidoglycan (PG) sacculus is an essential bacterial macromolecule that protects the cell from bursting due to its turgor and maintains the shape of the cell (Vollmer and Bertsche, 2008; Typas *et al.*, 2012). PG is composed by glycan chains connected by short peptides. The PG from different species varies in the structure of the peptides and presence of secondary modifications (Vollmer *et al.*, 2008). PG precursors are synthesized inside the cell and equipped with a carrier lipid for transport across the membrane prior to their polymerization at the outer leaflet of the cytoplasmic membrane (Barreteau *et al.*, 2008). The universal bacterial carrier lipid is undecaprenyl phosphate (C_55_-*P*), which is synthesized in two steps. First, UppS uses farnesyl pyrophosphate (C_15_-*PP*) and eight isopentenyl pyrophosphate (C_5_-*PP*) molecules to produce the diphosphate form of the carrier lipid (C_55_-*PP*), which is then dephosphorylated to C_55_-*P* by membrane embedded phosphatases (UppP, or PAP2-type phosphatases) (Manat *et al.*, 2014).

The final precursor for PG synthesis is lipid II, the GlcNAc-MurNAc(pentapepide) building block linked to C_55_-*PP*. Lipid II is synthesized in two steps at the inner leaflet of the cytoplasmic membrane from UDP-MurNAc-pentapeptide, UDP-GlcNAc and C_55_-*P* by the enzymes MraY and MurG (Bouhss *et al.*, 2008). PG glycosyltransferases (GTases) polymerize lipid II at the outer leaflet of the membrane to glycan chains. This reaction releases C_55_-*PP* which is dephosphorylated for new rounds of precursor synthesis and transport.

Peptidoglycan synthesis is a prime target for antibacterial compounds and enzymes. Bacteria and higher organisms often produce antibacterial compounds to target competing bacteria and invading pathogens, respectively (Malanovic and Lohner, 2016). Bacterial competition is particularly fierce in dense populations such as biofilms and soil communities. Whilst the group of actinomycetes are known for their capability to secrete a repertoire of small metabolites that often show antibacterial activity, many Gram-negative bacteria utilize sophisticated type VI secretion systems to target adjacent bacterial cells by antimicrobial enzymes (Russell *et al.*, 2011; 2012 and 2014). Another type of bacterial toxins are colicins, which are secreted by certain strains of *Escherichia coli* (Cascales *et al.*, 2007). Colicins use energized nutrient uptake systems to enter the periplasm of susceptible strains of *E. coli*. Most colicins kill the target cell by inserting into the cytoplasmic membrane to form pores (Braun and Patzer, 2013). An exception is colicin M, which has a phosphatase activity against lipid II, cleaving the essential peptidoglycan precursor to disaccharide pyrophosphate and undecaprenol (El Ghachi *et al.*, 2006).

More recently, it was shown that some Gram-positive species use a type VII secretion system to target other bacteria (Cao *et al.*, 2016). So far the best example is *Streptococcus intermedius*, which uses a type VII secretion system to deliver an antibacterial toxin, TelC, to target bacteria (Whitney *et al.*, 2017). TelC was shown to degrade lipid II and C_55_-*PP* to release disaccharide pentapeptide and pyrophosphate, respectively, and undecaprenol. *S. intermedius* also produces the immunity protein TipC, which inactivates TelC by direct interaction to prevent the lysis of the toxin-producing cell (Whitney *et al.*, 2017). In this methods paper, we provide a detailed description of the TLC and HPLC methods that established the degradation of lipid II and C_55_-*PP* by TelC (Whitney *et al.*, 2017). These methods can be generally used to assess the activity and specificity of phosphatases against membrane-bound bacterial cell wall precursors.

## Materials and Reagents

1. Pipette tips (STARLAB, catalog numbers: S1111-3700, S1113-1700, S1111-6701)
2. 1.5 ml micro-tubes (SARSTEDT, catalog number: 72.690.001)
3. Aluminium HPTLC silica gel 60 plates, 20 x 20 cm, without fluorescent indicator (Merck, catalog number: 1.05547.0001)
4. Glass vials (Soda glass, w/o rim, round bottom, 40 x 8 x 0.8-1.0 mm) (VWR, catalog number: 212-0011)
5. pH indicator strips (Merck, catalog number: 1.09531.0001)
6. HPLC vials (Agilent Technologies, catalog number: 5182-0553)
7. Vial inserts, 400 μl, glass, flat bottom (Agilent Technologies, catalog number: 5181-3377)
8. Hypodermic needles (FINE-JECT^®^ for single use) (VWR, catalog number: 613-2022)
9. 2 ml micro-tubes (SARSTEDT, catalog number: 72.695.500)
10. MF-Millipore membrane filter 0.22 μm, mixed cellulose esters (Merck, catalog number: GSWP04700)
11. Enzyme of interest/putative phosphatase
12. Potassium chloride (KCl) (Analytical Reagent Grade) (Fisher Scientific, CAS number: 7447-40-7)
13. Triton X-100 (Roche Diagnostics, catalog number: 10789704001)
14. Lipid II (Lys version) (gift from Eefjan Breukink, University of Utrecht) (Egan *et al.*, 2015)
  *Note: Lipid II can be produced and purified by previously reported methods (Brötz et al., 1995; Qiao et al., 2017)*.
15. Scintillation cocktail ProFlow G+ (Meridian Biotechnologies, catalog number: ProFlow G+)
  *Note: Used together with the radioactivity flow-through detector*.
16. Calcium chloride anhydrous (CaCl_2_) (Melford Laboratories, catalog number: C1103)
17. Undecaprenyl monophosphate diammonium salt (Larodan, catalog number: 62-1055)
18. Magnesium chloride hexahydrate (MgCl_2_·6H_2_O) (VWR, catalog number: 25108.260)
19. Farnesyl pyrophosphate ammonium salt (Sigma-Aldrich, catalog number: F6892)
20. Isopentenyl pyrophosphate triammonium salt solution (Sigma-Aldrich, catalog number: I0503)
21. Undecaprenyl pyrophosphate synthase (UppS) from *E. coli*, purified as described in Pan *et al.*, 2000
22. Undecaprenol (Larodan, catalog number: 60-1055)
23. n-Butanol (Honeywell International, catalog number: 537993)
24. Pyridine, anhydrous 99.8% (Sigma-Aldrich, catalog number: 270970)
25. Iodine (Sigma-Aldrich, catalog number: I3380)
26. [^14^C]GlcNAc-labeled lipid II (Lys version) (gift from Eefjan Breukink, University of Utrecht) (Egan *et al.*, 2015)
27. Chloroform (Sigma-Aldrich, catalog number: 32211-M)
28. Methanol (Fisher Scientific, catalog number: 10284580)
29. Sodium chloride (NaCl) (VWR, catalog number: 27810.295)
30. Methanol (CHROMASOLV™, gradient grade, for HPLC, ≥ 99.9%) (Honeywell International, Riedel-de Haën™, catalog number: 34885)
31. Peptidoglycan synthase PBP1B and its cognate activator LpoB proteins from *E. coli*, purified as described in Bertsche *et al.* (2006) and Egan *et al.* (2014)
32. Sodium borohydride (Merck, catalog number: 1.06371.0100)
33. Milli Q quality water (ddH_2_O)
34. Ammonium hydroxide (Honeywell International, catalog number: 05003)
35. Acetic acid > 99.8% (Sigma-Aldrich, catalog number: 33209-M)
36. 4-(2-Hydroxyethyl)-1-piperazineethanesulfonic acid (HEPES) (VWR, catalog number: 441485H)
37. Potassium hydroxide (KOH) (Sigma-Aldrich, catalog number: P5958-500G)
38. Sodium hydroxide (NaOH) (VWR, catalog number: 28245.298)
39. Sodium hydroxide for HPLC (semiconductor grade, 99.99% trace metals basis) (Sigma-Aldrich, catalog number: 306576)
40. ortho-Phosphoric acid, 85-90% (HPLC) (Honeywell International, Fluka™, catalog number: 79606)
41. Boric acid (99.97% trace metals basis) (Sigma-Aldrich, catalog number: 339067)
42. Muramidase cellosyl (provided by Höchst AG, Frankfurt, Germany)
  *Note: Alternatively, the muramidase mutanolysin (Sigma-Aldrich, catalog number: M9901) can be used*.
43. Sodium azide (NaN_3_) (Sigma-Aldrich, catalog number: S2002)
44. Mobile phase (see Recipes)
45. Undecaprenol and undecaprenyl monophosphate diammonium salt (see Recipes)
46. 6 M pyridinium acetate (see Recipes)
47. n-Butanol/pyridinium acetate pH 4.2 (see Recipes)
48. HEPES/KOH stock solution (1 M, pH 7.5) (see Recipes)
49. HEPES/NaOH stock solution (1 M, pH 7.5) (see Recipes)
50. Sodium phosphate buffer (80 mM, pH 4.8) (see Recipes)
51. Muramidase cellosyl (0.5 μg μl^-1^) (see Recipes)
52. Sodium borate (0.5 M, pH 9.0) (see Recipes)
53. HPLC buffer A (see Recipes)
54. HPLC buffer B (see Recipes)

## Equipment

1. Pipettes (Gilson, catalog numbers: F167300 and F167500)
2. AccuTherm Microtube Shaking Incubator (Labnet International, model: AccuTherm™, catalog number: I-4002-HCS)
3. Vacuum concentration system (Labogene, model: MaxiVac Alpha)
4. Bench top centrifuge, for example Accuspin Micro 17 microcentrifuge (Fisher Scientific, model: accuSpin™ Micro 17, catalog number: 75002460)
5. Chemical fume hood
6. TLC developing chamber (VWR, catalog number: 552-0363)
7. Proheat^®^ heat gun (Sigma-Aldrich, catalog number: Z673722-1EA)
8. Small beaker (Petri dish, Steriplan^®^) (VWR, catalog number: 391-2840)
9. Dry bath (Digital Dry Bath, Labnet International, catalog number: D1100-230V)
10. HPLC apparatus (Agilent Technologies, model: 1200 Series) with flow detector for radioactivity (LabLogic Systems, model: Beta-RAM 5)
11. ProntoSIL 120-3-C18AQ3um 250 x 4.6 mm HPLC column (Bischoff Chromatography, catalog number: 2546F184PS030)
12. IKA RH basic 2 magnetic stirrer (IKA, catalog number: 0003339002)
13. Vortex (IKA, model: Minishaker MS2)
14. pH meter (Cole-Parmer, Jenway, model: 3510, catalog number: 351001)
15. Incubator (Genlab, catalog number: INC/100/DIG)
16. Brown glass vials (Fisher Scientific, catalog number: 11531474)

**Part I: Thin-layer chromatography assay**

## Procedure

**Figure 1:**
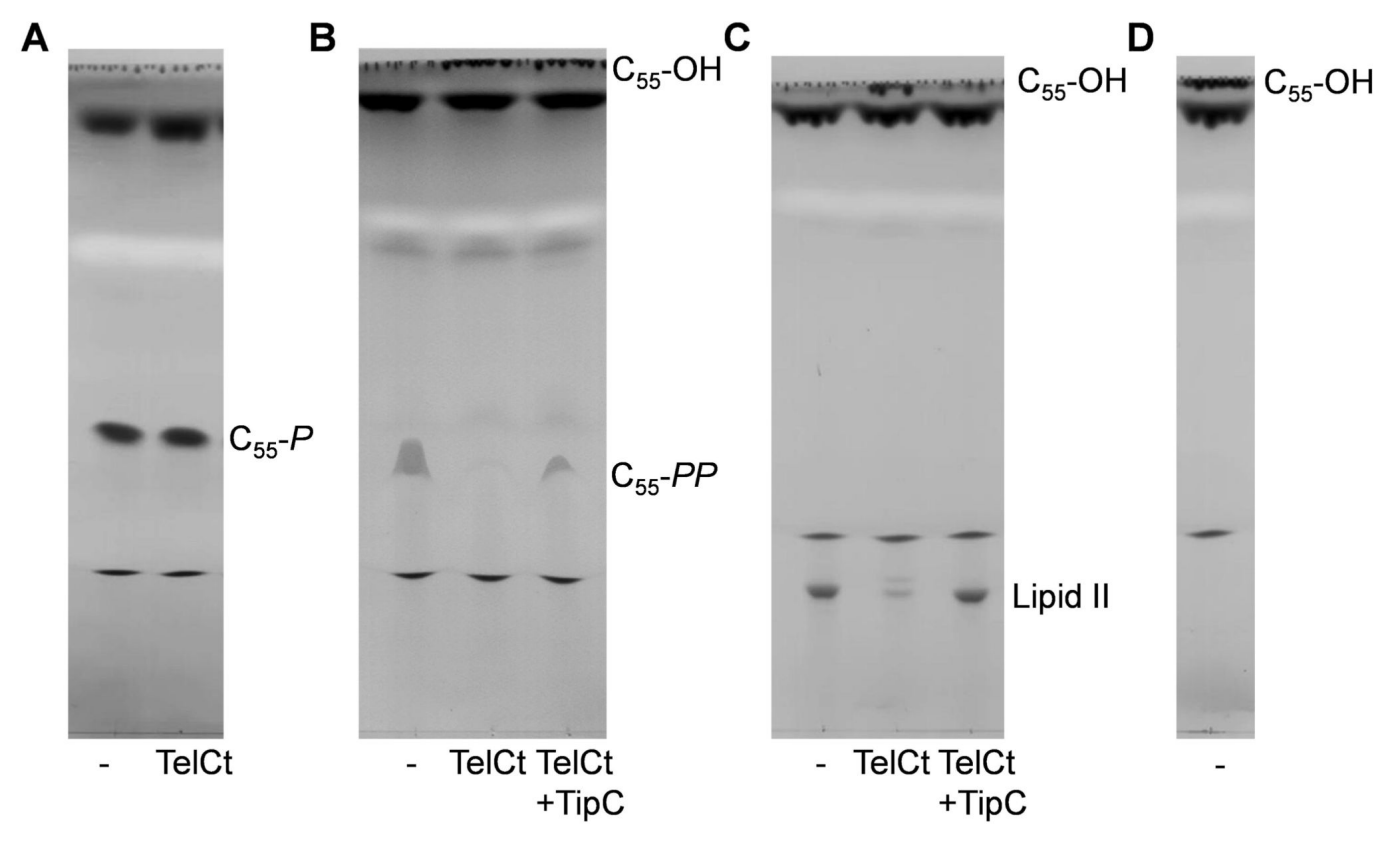
Lipid II and C_55_-*PP*, but not C_55_-*P*, are substrates of TelCt. Thin-layer chromatography analysis of the products obtained in reactions of TelCt (toxin domain of TelC) or TelCt-TipC with (A) C_55_-*P*, (B) C_55_-*PP* or (C) lipid II. D. C_55_-OH migrates at the solvent front. Control samples (-) contained no protein. TelCt was active against lipid II and C_55_-*PP* and was inhibited by its immunity protein TipC. The figure was adopted from Whitney *et al.* (2017). *Note: Only lipid II and undecaprenyl monophosphate will appear as sharp bands. Due to its amphiphilic nature undecaprenyl pyrophosphate will appear as a crescent-shaped band between lipid II and undecaprenyl phosphate. The hydrophobic undecaprenol will always migrate at the solvent front, but will be visible after iodine staining*.

- Enzymatic digestion of lipid II or undecaprenyl pyrophosphate
  *Note: All reactions are carried out in 1.5 ml microtubes and incubated using a microtube shaking incubator at 800 rpm*.
  1. Reactions are carried out in a final volume of 50 μl and set up as described below for each substrate. All enzyme substrates are dried under vacuum and subsequently solubilized in the reaction mixture.
    *Note: Take into account the constituents present in the storage buffer of the assayed proteins to calculate the buffer mixture*.
    - Lys-type lipid II
      Prepare enzyme reactions with final concentrations of 30 mM HEPES/KOH pH 7.5, 150 mM KCl, 0.1% (w/v) Triton X-100, 40 µM lipid II (L-Lys). Add 2 µM phosphatase (*e.g*., TelCt), phosphatase-inhibitor complex (*e.g*., TelCt-TipC complex) or no enzyme (control) and incubate for 90 min at 37 °C.
    - Undecaprenyl monophosphate
      Prepare enzyme reactions with final concentrations of 20 mM HEPES/KOH pH 7.5, 150 mM KCl, 1 mM CaCl_2_, 0.1% (w/v) Triton X-100, 100 µM undecaprenyl monophosphate. Add 2 µM phosphatase (*e.g*., TelCt) or no enzyme (control) and incubate for 90 min at 37 °C.
    - Undecaprenyl pyrophosphate synthesis coupled to the degradation by TelCt
      Prepare enzyme reactions with final concentrations of 20 mM HEPES/KOH pH 7.5, 50 mM KCl, 0.5 mM MgCl_2_, 1 mM CaCl_2_, 0.1% (w/v) Triton X-100, 40 µM farnesyl pyrophosphate, 400 µM isopentenyl pyrophosphate, 10 µM UppS. Add 2 µM phosphatase (*e.g*., TelCt), phosphatase-inhibitor complex (*e.g*., TelCt-TipC complex) or no enzyme (control) and incubate for 5 h at 25 °C, followed by an additional incubation for 90 min at 37 °C.
    - Undecaprenol
      Prepare enzyme reactions with final concentrations of 30 mM HEPES/KOH pH 7.5, 150 mM KCl, 0.1% (w/v) Triton X-100 and 100 µM undecaprenol. Incubate for 90 min at 37 °C.
  2. Terminate the reactions by adding 50 µl of n-butanol/pyridine acetate (2:1) pH 4.2.
  3. Vortex for 1 min and centrifuge for 3 min at 17,000 *x g* using a bench-top centrifuge to separate the organic phase (n-butanol) from the aqueous phase (pyridine-acetate, water).
    *Note: This step is essential to extract hydrophobic (lipid II, C_55_-P, C_55_-OH) and amphiphilic (C_55_-PP and Triton X-100) substances from the mixture. These substances will be found in the upper, organic phase which will contribute to 1/3 of the total volume*.
- Thin layer chromatography
  *Note: All steps are carried out in a chemical fume hood at room temperature if not indicated otherwise. The basic procedure for thin layer chromatography is shown in the published movie (Cockburn and Koropatkin, 2015)*.
  1. Pour the mobile phase into the developing chamber and adjust the solvent level to 1 cm.
  2. Close the chamber with the lid and allow for saturation of the gaseous phase with solvent (60 min).
  3. Incubate the TLC plate for at least 1 h at 60 °C to remove any humidity left from storage.
  4. Use a pencil to draw a line 1.5 cm from the bottom of the plate and mark sample spots. Sample spots are separated by 2 cm, and the distance from the outer spots to the edge of the plate should be at least 4 cm.
  5. Load the complete organic phase (upper phase, see Step A3) in 10 µl aliquots on the sample spots. After the addition of each aliquot, the spot is dried with a heat gun. Alternatively, the plate is left under a fume hood for each drying step.
    *Note: It is important that the lower (aqueous) phase is not transferred on the plate, as this will result in smearing of the spots. When using a heat gun, it is important to not overheat the spots, as this can lead to degradation of compounds and additional bands*.
  6. Place the TLC carefully in the developing chamber such that the solvent does not reach the spots. Optimally, there should be a distance of 0.5 cm between the solvent level and the pencil line.
  7. The TLC plate is incubated in the chamber with the lid on until the solvent front reaches 4/5 of the plate length, which takes 1.5-2 h.
- Staining
  1. Remove the plate from the chamber and dry it with a heat gun. The plate should be completely dried to avoid the appearance of solvent bands during staining.
  2. Place a small beaker with iodine in the chamber and put the lid on. Saturation will take 20-30 min at room temperature.
  3. Place the plate in the development chamber saturated with iodine vapor and incubate it until the spots are clearly visible (20 min-1 h).
  4. Representative examples of each reaction are shown in Figure 1.

## Data analysis

Take a high-resolution picture and determine the retention factor (R_f_) using commercially available programs (*e.g.,* ImageJ). Bands present in control reactions serve as a standard.

$$R_{f}=\frac{\text{distance}\;\text{of}\;\text{the}\;\text{substance}\;\text{zone}\;\text{from}\;\text{the}\;\text{sample}\;\text{origin}\;[\text{mm}]}{\text{solvent}\;\text{front}\;\text{migration}\;\text{distance}\;[\text{mm}]}$$

*Note: The distance is measured from the application line to the middle of the substance spots. For asymmetric spots (here: undecaprenyl pyrophosphate) measure the distance between the application line and lowest point of the spot. Spots in reaction mixtures should have similar R_f_ values, shape and color as spots derived from the standard compounds*.

**Part II: High performance liquid chromatography assay**

## Procedure

**Figure 2:**
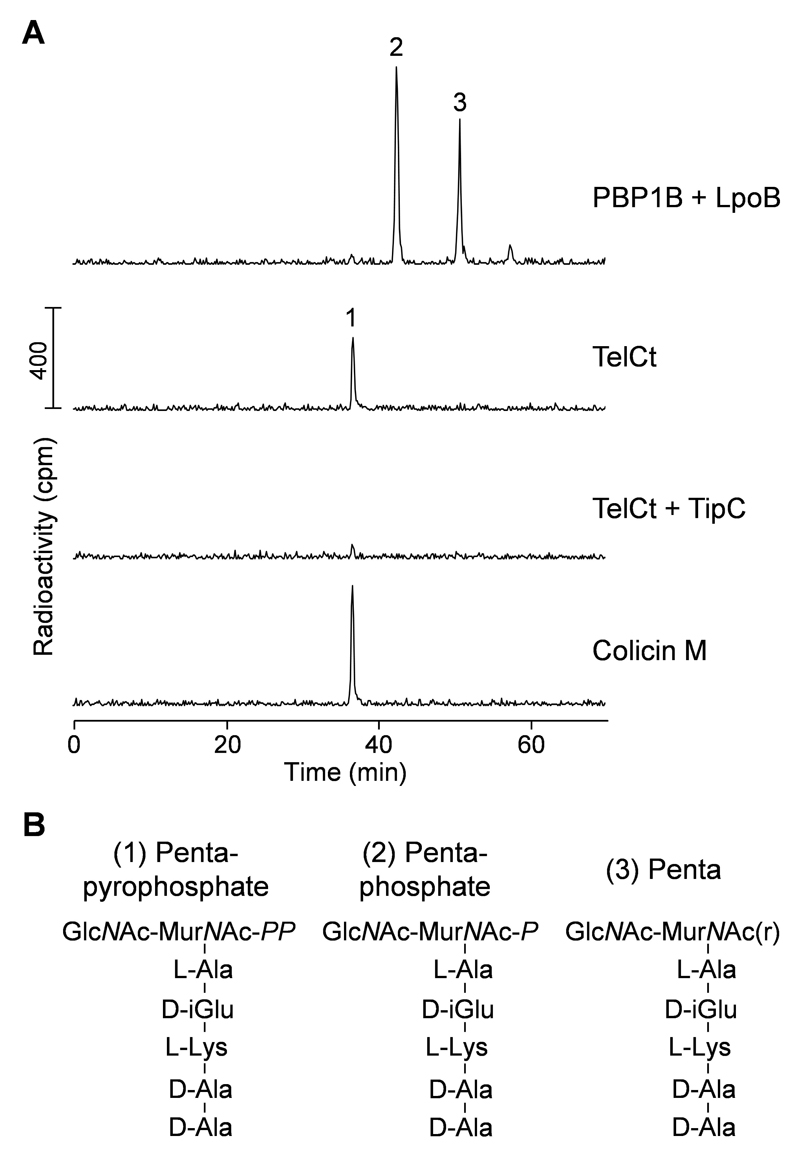
TelCt cleaves lipid II between undecaprenyl and pentapeptide-pyrophosphate. A. HPLC chromatograms of the radiolabeled products resultant from reactions containing Lys-Lipid II and the indicated proteins. PBP1B + LpoB reaction was further digested with cellosyl and reduced with sodium borohydride. B. Proposed structures of the main products (peaks 1-3 in panel A) of each reaction. GlcNAc, N-acetylglucosamine; MurNAc-*PP*, N-acetylmuramic acid pyrophosphate; MurNAc-*P*, N-acetylmuramic acid phosphate; MurNAc(r), N-acetylmuramitol; L-Ala, L-alanine; L-Lys, L-lysine; D-iGlu, D-isoglutamic acid; D-Ala, D-alanine. The figure was adopted from Whitney *et al.* (2017).

- Lipid II reactions for HPLC assay
  *Note: A control reaction, containing the peptidoglycan synthase PBP1B and its cognate activator LpoB, is assayed to polymerize lipid II into short glycan chains with C_55_-PP at the terminal MurNAc residue*.
  1. Dry 10,000 dpm (~1 nmol) of [^14^C]GlcNAc-labeled lipid II-Lys, stored in chloroform/methanol (1:1), in a glass vial using a vacuum.
  2. Resuspend the lipid II in 5 μl of 0.2% (w/v) Triton X-100 and vortex for 10 sec at 1,800 rpm.
  3. Prepare in a 1.5 ml microtube a reaction buffer mixture with final concentrations of 15 mM HEPES/NaOH, pH 7.5, 10 mM MgCl_2_, 150 mM NaCl, 0.023% (w/v) Triton X-100 and 0.4 mM CaCl_2_ (the PBP1B-LpoB control reaction did not contain CaCl_2_) in a total reaction volume of 100 μl.
    *Note: Take into account the constituents present in the storage buffer of the assayed proteins to calculate the buffer mixture*.
  4. Add 2 μM phosphatase (*e.g*., TelCt or Colicin M) or phosphatase-inhibitor complex (TelCt-TipC) to the reaction buffer. For a control sample add 0.75 μM PBP1B and 1.5 μM LpoB to the reaction buffer.
  5. Add the reaction mixture to the resuspended lipid II and incubate it for 60 min in a microtube shaking incubator at 37 °C with shaking (800 rpm).
  6. Spin down the condensation using a microcentrifuge.
  Reactions with phosphatases (TelCt, TelCt-TipC or Colicin M) are processed as follows:
  7. Adjust the pH of the sample to 3.5-4.0 using 20% phosphoric acid and pH indicator stripes.
    *Note: Measure the pH by putting 0.3 μl sample onto the pH indicator stripe*.
  8. Centrifuge the sample in a microcentrifuge for 15 min at maximum speed and room temperature. Transfer the supernatant into an HPLC vial containing a 400 µl vial insert.
  The control reaction with PBP1B-LpoB requires additional steps to digest the peptidoglycan with a muramidase and reduce the resulting unphosphorylated muropeptides. After Step A6 the reaction must be processed as follows:
  9. Incubate samples for 5 min at 100 °C using a dry bath, then spin down the condensation using a microcentrifuge.
  10. Let the samples cool down at room temperature for 2 min.
  11. Add 30 μl of cellosyl buffer (80 mM sodium phosphate, pH 4.8) and 10 µl of 0.5 µg µl^-1^ cellosyl (or mutanolysin) to the sample.
  12. Incubate the samples for 70 min in a microtube shaking incubator at 37 °C with shaking (800 rpm).
  13. Spin down the condensation using a microcentrifuge.
  14. Boil the reaction for 10 min at 100 °C on a dry bath and centrifuge the sample using a microcentrifuge for 15 min at maximum speed and room temperature.
  15. Punch a hole in the lid of a new 2 ml microcentrifuge tube using a needle.
    *Note: The hole will allow releasing the H_2_ gas produced during the reduction step*.
  16. Transfer the supernatant to the 2 ml microcentrifuge tube.
  17. Reduce the muropeptides by adding 100 μl of 0.5 M sodium borate, pH 9.0 and a tip of a spatula of solid sodium borohydride (*ca.* 1 mg).
  18. Incubate the sample for 30 min at room temperature in a microcentrifuge at 4,700 *x g* to prevent spillage due to gas bubbles.
  19. Adjust the pH of the sample to 3.5-4.0 using 20% phosphoric acid and pH indicator stripes.
    *Note: Measure the pH by putting 0.3 μl of sample onto the pH indicator stripe*.
  20. Centrifuge the sample in a microcentrifuge for 15 min at maximum speed and room temperature. Transfer the supernatant into an HPLC vial containing a 400 µl vial insert.
- Detection of lipid II products by HPLC
  System and set up conditions:
  HPLC connected to a radioactivity flow-through detector
  C18 reversed-phase column
  Flow rate: 0.5 ml min^-1^
  Column temperature: 55 °C
  1. Wash with 100% methanol for 20 min at room temperature.
  2. Increase column temperature to 55 °C.
  3. Start a linear gradient for 30 min from 100% methanol to 100% Milli Q water, holding 100% Milli Q water for further 20 min.
  4. Wash with HPLC buffer B for 20 min and equilibrate the column with HPLC buffer A for 40 min.
  5. Do a buffer run following the same method used for the samples of interest (Steps B6-B9) but without injecting any sample.
  6. Inject the sample (leave 20 µl of the total reaction volume in the vial insert) and flush the injection loop with HPLC buffer A for 2 min.
  7. Start a linear elution gradient for 60 min from 100% HPLC buffer A to 50% HPLC buffer B, holding 50% HPLC buffer B for further 10 min.
  8. Re-equilibrate the column with 100% HPLC buffer A for 30 min.
  9. Inject the next sample.
  Representative HPLC chromatograms of each sample are shown in Figure 2.

## Data analysis

1. Each reaction should be assayed in triplicate.
2. The produced muropeptides are identified based on their retention time, using the software provided with the HPLC system (*e.g*., Laura™, LabLogic Systems Ltd).
3. If needed, the phosphatase product can be verified by mass spectrometry. For this, use 16 nmol of non-radioactive lipid II (Lys version) as substrate to perform the reaction as described above, using a UV detector set at 205 nm, and collect the product fraction. Dry the collected fraction in a SpeedVac and store it at -20 °C until mass spectrometry analysis as reported (Bui *et al.*, 2009).
  *Note: The products can be collected either manually or using the HPLC collector module*.

## Recipes

*Note: Unless otherwise indicated, all stock solutions are prepared using Milli Q water*.

1. Mobile phase (according to Rick *et al.*, 1998)
  Mix components in the following order under gentle stirring:
  10 ml water
  1 ml 36% ammonium hydroxide
  48 ml methanol
  88 ml chloroform
  Store in a brown glass bottle
  *Note: Chloroform should be added step-wise and slowly to prevent phase separation*.
2. Undecaprenol and undecaprenyl monophosphate diammonium salt
  Prepare 1 mM solutions in chloroform-methanol (2:1, v:v)
  Store in brown glass vials at -20 °C
3. 6 M pyridinium acetate
  Mix 51.5 ml glacial acetic acid with 48.5 ml of pyridine
4. n-Butanol/pyridinium acetate pH 4.2 (according to van Heijenoort *et al.*, 1992)
  Mix 50 ml n-butanol with 25 ml 6 M pyridinium acetate
5. HEPES/KOH stock solution (1 M, pH 7.5)
  Dissolve 23.83 g N-(2-hydroxyethyl)piperazine-N’-(2-ethanesulfonic acid) (HEPES) in 90 ml of Milli Q water
  Adjust pH to 7.5 with potassium hydroxide (1 M)
  Adjust to a final volume of 100 ml
6. HEPES/NaOH stock solution (1 M, pH 7.5)
  Dissolve 23.83 g N-(2-hydroxyethyl)piperazine-N’-(2-ethanesulfonic acid) (HEPES) in 90 ml of Milli Q water
  Adjust pH to 7.5 with sodium hydroxide (1 M)
  Adjust to a final volume of 100 ml
7. Sodium phosphate buffer (80 mM, pH 4.8)
  Dissolve 0.64 g sodium hydroxide (for HPLC) in 150 ml Milli Q water
  Adjust pH to 4.8 with phosphoric acid (85% and 20%)
  Adjust to a final volume of 200 ml
8. Muramidase cellosyl (0.5 μg μl^-1^)
  Dissolve 5 mg of freeze-dried muramidase cellosyl stock in 10 ml of 20 mM sodium phosphate, pH 4.8
  Aliquot in microtubes and store at -20 °C
9. Sodium borate (0.5 M, pH 9.0)
  Dissolve 3.09 g boric acid in 75 ml Milli Q water
  Adjust pH to 9.0 with sodium hydroxide (10 M)
  Adjust to a final volume of 100 ml
10. HPLC buffer A (50 mM sodium phosphate, pH 4.31 with 10 μl of 10% sodium azide per liter of buffer)
  Dissolve 4 g sodium hydroxide (for HPLC) in 1,900 ml Milli Q water
  Adjust pH to pH 4.31 with phosphoric acid (85% and 20%)
  Adjust to a final volume of 2 L
  Filter the buffer using a 0.22 μm filter
  Add 20 μl of 10% sodium azide
11. HPLC buffer B (75 mM sodium phosphate, pH 4.95, 15% v/v methanol)
  Dissolve 6 g sodium hydroxide (for HPLC) in 1,500 ml Milli Q water
  Adjust pH to pH 4.95 with phosphoric acid (85% and 20%)
  Adjust to a final volume of 1.7 L
  Filter the buffer through a 0.22 μm filter
  Add 300 ml of methanol for HPLC
